# Trimetazidine ameliorates sunitinib-induced cardiotoxicity in mice via the AMPK/mTOR/autophagy pathway

**DOI:** 10.1080/13880209.2019.1657905

**Published:** 2019-09-23

**Authors:** Yi Yang, Na Li, Tongshuai Chen, Chunmei Zhang, Lingxin Liu, Yan Qi, Peili Bu

**Affiliations:** Department of Cardiology, The State and Shandong Province Joint Key Laboratory of Translational Cardiovascular Medicine, Chinese National Health Commission and Chinese Academy of Medical Sciences, Qilu Hospital of Shandong University, Jinan, Shandong, China

**Keywords:** Hypertension, left ventricular dysfunction, cardiomyocyte, tyrosine kinase inhibitor

## Abstract

**Context:** Sunitinib (SU) is a multi-targeted tyrosine kinase inhibitor anticancer agent whose clinical use is often limited by cardiovascular complications. Trimetazidine (TMZ) is an anti-angina agent that has been demonstrated cardioprotective effects in numerous cardiovascular conditions, but its potential effects in SU-induced cardiotoxicity have not been investigated.

**Objective:** This study investigates the effect of TMZ in sunitinib-induced cardiotoxicity *in vivo* and *in vitro* and molecular mechanisms.

**Materials and methods:** Male 129S1/SvImJ mice were treated with vehicle, SU (40 mg/kg/d) or SU and TMZ (20 mg/kg/d) via oral gavage for 28 days, and cardiovascular functions and cardiac protein expressions were examined. H9c2 cardiomyocytes were treated with vehicle, SU (2–10 μM) or SU and TMZ (40–120 μM) for 48 h, and cell viability, apoptosis, autophagy, and protein expression was tested.

**Results:** SU induces hypertension (systolic blood pressure [SBP] + 28.33 ± 5.00 mmHg) and left ventricular dysfunction (left ventricular ejection fraction [LVEF] − 11.16 ± 2.53%) in mice. In H9c2 cardiomyocytes, SU reduces cell viability (IC_50_ 4.07 μM) and inhibits the AMPK/mTOR/autophagy pathway (*p* < 0.05). TMZ co-administration with SU reverses SU-induced cardiotoxicity in mice (SBP − 23.75 ± 4.69 mmHg, LVEF + 10.95 ± 3.317%), alleviates cell viability loss in H9c2 cardiomyocytes (*p* < 0.01) and activates the AMPK/mTOR/autophagy pathway *in vivo* (*p* < 0.001) and *in vitro* (*p* < 0.05).

**Discussion and conclusions:** Our results suggest TMZ as a potential cardioprotective approach for cardiovascular complications during SU regimen, and potentially for cardiotoxicity of other anticancer chemotherapies associated with cardiomyocyte autophagic pathways.

## Introduction

Modern advances in anticancer chemotherapies markedly optimize survival and outlook of cancer patients. Unfortunately, lack of enough specificity of their tumor-killing efficacy leads to cardiovascular adverse effects, which heavily cripple life quality of cancer patients, and sometimes cancer survivors end up dying of cardiac events. Sunitinib (SU) is a tyrosine kinase inhibitor antineoplastic agent clinically used for the treatment of cancers including renal cell carcinoma and gastrointestinal stromal tumors, but its clinical application is hampered by potential cardiotoxicity (Reichardt et al. 2015). In a recent multicenter study of SU in 71 thyroid carcinoma patients, the incidence of cardiac events, including cardiac insufficiency, cardiac infarction and left ventricular ejection fraction (LVEF) decrease, mitral insufficiency, pericardial effusion and tachycardia was 14.1% (Ravaud et al. 2017). This calls for developing strategies for the prevention and treatment of SU-induced cardiotoxicity that specifically act cardiovascular-wise and do not compromise its tumour-killing potency.

Trimetazidine (TMZ) is a conventionally used drug for angina pectoris. TMZ acts by selectively inhibiting mitochondrial long-chain 3-ketoacyl-CoA thiolase (HADHA), a key enzyme for the β-oxidation of fatty acids, and in turn, inhibits β-oxidation of fatty acids and promotes glucose utilization, improving cardiac energy metabolism (Lopatin et al. 2016). Although primarily used for coronary artery diseases, TMZ has also shown cardioprotective effects in a range of other cardiovascular diseases, including non-ischemic cardiomyopathy, sepsis, anticancer drug-induced cardiotoxicity, diabetic cardiomyopathy and contrast-induced nephropathy (Zou et al. 2017). To date, TMZ efficacy in anticancer drug-induced cardiotoxicity has mainly focused on anthracycline-induced cardiotoxicity. TMZ has been demonstrated to impede anthracycline-induced lipid peroxidation and inflammation and exert cardioprotective effects (Tallarico et al. 2003; Mele et al. 2016). Nevertheless, SU injures the myocardium via mechanisms different from anthracyclines, and potential effects of TMZ in SU-induced cardiotoxicity have not been investigated.

In this study, we sought to determine the potential effects of TMZ in SU-induced cardiotoxicity. Using both the mouse model of sunitinib cardiotoxicity and the *in vitro* H9c2 cardiomyocyte model of SU toxicity and SU-TMZ co-administration, we show that TMZ reverses SU-induced hypertension and left ventricular dysfunction (LVD) in mice, and alleviates SU-induced H9c2 cardiomyocyte viability loss via the AMPK/mTOR/autophagy pathway.

## Materials and methods

### Animal and treatments

Wild-type 129S1/SvImJ mice were purchased from the Jackson Laboratory (Bar Harbor, ME, USA). Eighteen male 8-week-old mice were used in the study, and they were randomized into 3 groups of 6. The SU group received 40 mg/kg/day of SU (Selleckchem, Houston, TX, USA) via oral gavage for 28 days; SU-TMZ co-administration group received 40 mg/kg/day of SU and 20 mg/kg/day of TMZ (Servier, Suresnes, France) for 28 days, while control groups received daily administration of the vehicle of the corresponding volume. The 40 mg/kg/day dose of SU is the calculated dose for mice that corresponds to the clinical dose in humans (Chintalgattu et al. 2013). The 20 mg/kg/day dose of TMZ is the dose that was previously shown to exert significant cardioprotection in mice (Chen et al. 2016; Gong et al. 2018). Mice were housed in individually ventilated cages supplied with laboratory standard chow and water. All studies in animals were approved by the Animal Care and Use Committee of Shandong University and were conducted in accordance with the Guide for the Care and Use of Laboratory Animals, 8th edition (Washington Institute for Laboratory Animal Research 2011).

### Echocardiography

Echocardiography was performed with the VisualSonics Vevo 770 machine using a 30 MHz high-frequency transducer (Vevo 1100 system; VisualSonics, Toronto, Canada), according to previously described protocols (Gao et al. 2011). Mice were anesthetized lightly using isofluorane, shaved, and at least 3 M-mode measurements per mice were obtained. LVEF and fractional shortening (FS) were calculated to represent left ventricular functions.

### Blood pressures

Blood pressure parameters including systolic blood pressure (SBP), mean blood pressure (MBP) and diastolic blood pressure (DBP) were measured using a noninvasive tail-cuff system (BP2010A, Softron, Beijing, China). At least three measurements were recorded for each mouse and averaged.

### TUNEL staining

Mouse heart cryosections were stained using In Situ Cell Death Detection Kit, TMR red (12156792910, Roche, Basel, Switzerland) according to manufacturer’s protocols, and counterstained with DAPI to visualize nuclei. The images were photographed using confocal laser scanning microscopy (Zeiss, Oberkochen, Germany) and quantified using Image Pro Plus 6.0 software (Media Cybernetics, Sarasota, FL, USA). Measurements of at least three observations were averaged for TUNEL/DAPI ratio per mouse sample.

### Cell viability assay

Cell viability assays of H9c2 cardiomyocytes were conducted using Cell counting kit-8 (CCK-8, Dojindo, Kumamoto, Japan). H9c2 cells were planted onto 96-well and treated with SU or co-treated with SU and TMZ for 48 h, and then media were changed and cells were added with CCK-8 and incubated for 1 h. The Optical density absorbances at 450 nm were measured, and relative cell viability was calculated as viability versus control. IC50 of SU was calculated using Graphpad Prism 8.0.2 (Graphpad, San Diego, CA, USA). Each CCK-8 experiment was repeated at least 3 times.

### Immunoblotting analysis

Protein samples were obtained from both mouse ventricular homogenates and cultured H9c2 cardiomyocytes. The proteins (50 µg) were electrophoresed on 12.5% SDS-PAGE gels and transferred onto a polyvinylidene fluoride membrane using a wet transfer apparatus (Bio-Rad, Hercules, CA, USA). The membranes were incubated with primary antibodies, including LC3-I and -II, AMPK, p-AMPK, mTOR, p-mTOR and GAPDH overnight at 4 °C, washed, and then incubated with secondary antibodies labeled with horseradish peroxidase. The protein bands were analyzed using an ImageQuant LAS4000 chemiluminescence reader (GE, Boston, MA, USA). Each experiment was repeated at least 3 times.

### Statistical analysis

All statistical analyses were performed using Graphpad Prism 8.0.2 (Graphpad, San Diego, CA, USA). The data are expressed as the mean ± standard error of the mean (SEM), with center values defined as means. The Student’s two-tailed *t*-test was used to determine the significance of the differences between the compared values. Differences *p* < 0.05 were considered statistically significant.

## Results

### TMZ reverses SU-induced hypertension and LVD

**Figure 1.**
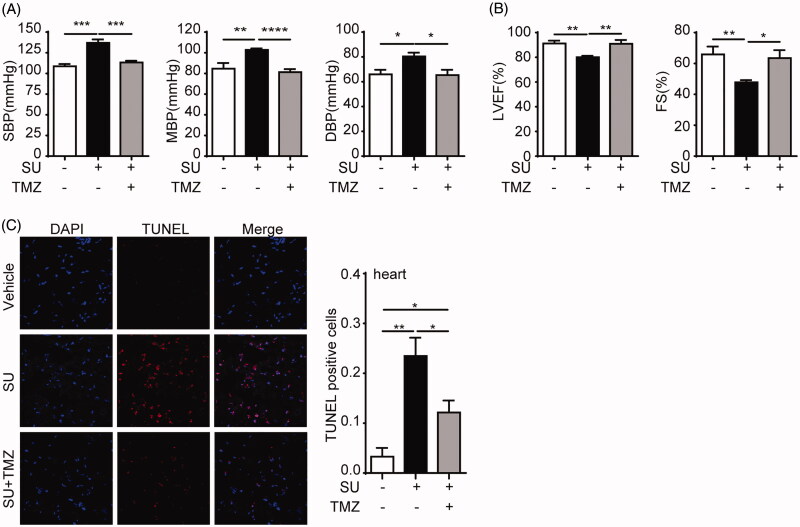
TMZ ameliorates SU-induced hypertension and LVD in mice. (A) Blood pressure parameters of vehicle-, SU- and SU-TMZ-treated mice (*n* = 6). (B) Left ventricular systolic function parameters of vehicle-, SU- and SU-TMZ-treated mice (*n* = 6). (C) Representative TUNEL assay of myocardial sections of vehicle-, SU- and SU-TMZ-treated mice and statistical analysis (*n* = 5, bar = 200 µm). (The Student’s two-tailed *t*-test was used, error bar = SEM, *p < 0.05, **p < 0.01, ***p < 0.001, ****p < 0.0001).

To evaluate the effects of TMZ on SU-induced cardiotoxicity, we treated mice with the vehicle, SU, or SU and TMZ for 28 days and examined cardiovascular parameters at the end of the experiment. Consistent with previous studies (Di Lorenzo et al. 2009; Ewer et al. 2014; Jacob et al. 2016; Narayan et al. 2017), SU-treated mice exhibited significantly elevated SBP, MBP, DBP, and lowered LVEF and FS, compared with vehicle-treated mice, indicating that SU triggered hypertension and LVD in mice (Figure 1(A,B)). Moreover, co-treatment of SU and TMZ completely reversed these effects (Figure 1(A,B)). Furthermore, TUNEL analysis showed that SU induced increased cell death rates in cardiomyocytes (Figure 1(C)). Once again, this increase in cell death was significantly alleviated by TMZ co-treatment (Figure 1(C)). These results indicate that TMZ co-treatment with SU ameliorates SU-induced hypertension, LVD, and significantly mitigates SU-induced cardiomyocyte death.

### TMZ ameliorates SU-induced autophagic inhibition

**Figure 2.**
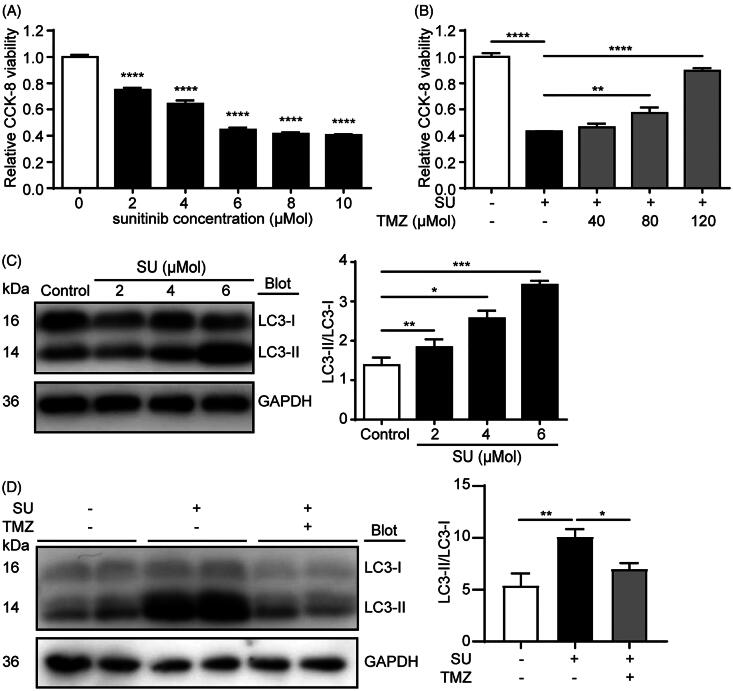
SU-induced H9c2 cardiomyocyte viability loss and autophagic inhibition are reversed by TMZ. (A) CCK-8 viability assay of H9c2 cardiomyocytes treated with vehicle or SU (*n* = 3). (B) CCK-8 viability assay of H9c2 cardiomyocytes treated with vehicle, SU (6 µM), and co-treated with SU and TMZ (*n* = 12). (C) Western blots of LC3-II/LC3-I in vehicle- and SU-treated H9c2 cardiomyocytes and statistical analysis (*n* = 6). (D) Western blots of LC3-II/LC3-I in vehicle-, SU- and SU-TMZ-treated H9c2 cardiomyocytes and statistical analysis (*n* = 6). (The Student’s two-tailed *t*-test was used, error bar = SEM, *p < 0.05, **p < 0.01, ***p < 0.001, ****p < 0.0001).

We next investigated the effects of SU or SU-TMZ co-treatment in cultured H9c2 cardiomyocytes. As shown by CCK-8 assay, 2–10 μM of SU caused significant cell viability declines in H9c2 cardiomyocytes, with IC_50_ of 4.065 μM (Figure 2(A)). Co-treatment of 6 μM SU and 80 μM or 120 μM TMZ significantly alleviated SU-induced viability decline, and 120 μM of TMZ restored cellular viability to up to mean relative viability of 89% (Figure 2(B)).

We then sought to explore mechanisms underlying these viability changes. Because apoptosis is the most common cause of cell death, we first analyzed apoptotic markers, including caspase-3 and cleave caspase-3 and Bax in vehicle- and SU-treated H9c2 cells. However, no significant alterations were found in these apoptotic markers between groups (Figure S1(A,B)), implying that apoptosis does not play an important role in SU-induced cardiomyocyte injuries.

Previously, autophagic inhibition has been shown to play a significant role in SU-induced cardiotoxicity (Zhao et al. 2010; Jacob et al. 2016; DeVorkin et al. 2017; Dyczynski et al. 2018; Thakur et al. 2018). Studies have shown that SU induces late-stage autophagic inhibition in both cancer cells and somatic tissues and cells, and that inhibiting autophagy enhances sensitivity to SU (Zhao et al. 2010; Jacob et al. 2016; DeVorkin et al. 2017; Dyczynski et al. 2018; Thakur et al. 2018). TMZ has been shown to promote autophagy in cardiac tissue and cardiomyocytes (Zhang et al. 2016; Zhong et al. 2017). This overlap of mechanisms prompted us to the hypothesis that TMZ may be potentially cardioprotective with the involvement of autophagy regulations. LC3 is a marker of autophagic vesicles. During autophagy, the autophagosome is fused with the lysosome, and LC3-I is converted to LC3-II and degraded within the autophagosome contents. Thus raised ratio of LC3-II/LC3-I indicates a more fluent autophagic flux, while the suppressed ratio of LC3-II/LC3-I suggests blockade in the autophagic flux, where fusion of autophagosome with the lysosome is impeded and cytoplasmic autophagosomes are accumulated. To test our hypothesis, we treated H9c2 cells with 2, 4 and 6 μM SU for 48 h and analyzed LC3-II/LC3-I ratios using western blot. Our results demonstrate that SU drastically increased LC3-II/LC3-I ratios in H9c2 cells, indicating that SU inhibited the autophagic flux (Figure 2(C)). Furthermore, co-treatment of 6 μM SU and 80 μM TMZ dramatically restored SU-induced LC3-II/LC3-I ratio increase (Figure 2(D)). Taken together, these results show that TMZ restores SU-induced cell viability decline, majorly via autophagic restoration.

### SU inhibits autophagy via AMPK/mTOR pathway

**Figure 3.**
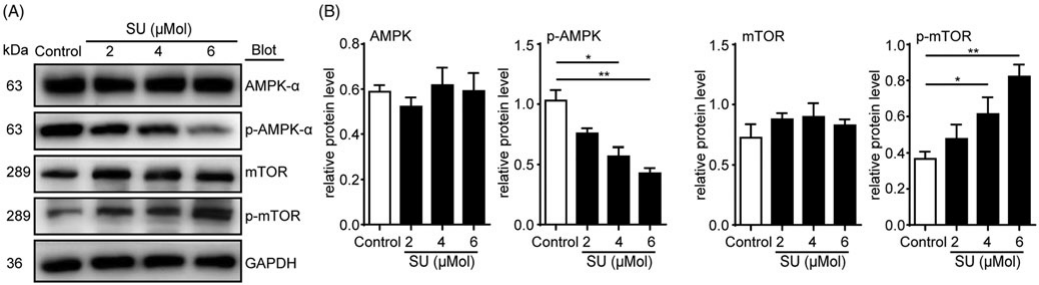
SU induces AMPK/mTOR activation inhibition in H9c2 cardiomyocytes. (A, B) Western blots of AMPK/mTOR pathway in vehicle- and SU-treated H9c2 cardiomyocytes and statistical analysis (*n* = 4). (The Student’s two-tailed *t*-test was used, error bar = SEM, *p < 0.05, **p < 0.01).

We next examined the possible molecular pathways that mediated SU-regulated H9c2 cell autophagy. The AMPK/mTOR axis is one of the classic core molecular regulators of autophagy (Klionsky et al. 2016). AMPK can be phosphorylated into its active form p-AMPK, which in turn may inhibit mTOR from activating into p-mTOR, and because p-mTOR is an autophagy inhibitor, AMPK inhibition of mTOR activation may, in turn, promote autophagy. We treated H9c2 cells with vehicle or 2, 4 and 6 μM SU and found that although SU did not alter total protein levels of AMPK and mTOR, 4 μM and 6 μM of SU was able to inhibit AMPK activation and enhance mTOR activation (Figure 3(A,B)). These findings implicate that the AMPK/mTOR axis may play an essential role in SU-induced cardiomyocyte autophagic inhibition.

### TMZ alleviates SU-induced autophagy via the AMPK/mTOR pathway

**Figure 4.**
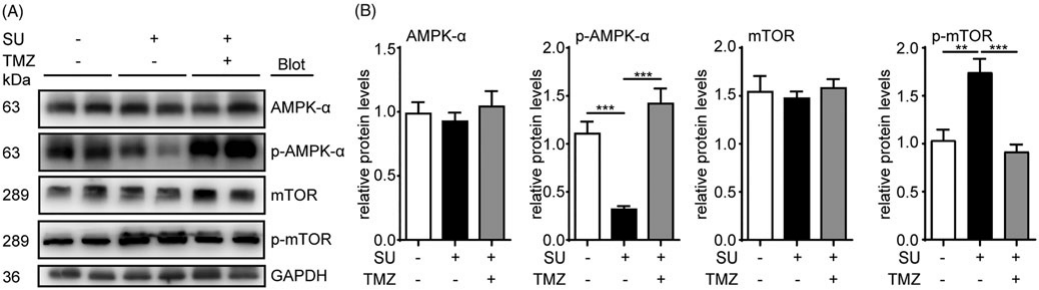
SU-induced AMPK/mTOR activation inhibition in mouse hearts is reversed by TMZ. (A, B) Western blots of AMPK/mTOR pathway in vehicle-, SU- and SU-TMZ-administrated mouse heart homogenates and statistical analysis (*n* = 5, 5, 5, 6, respectively). (The Student’s two-tailed *t*-test was used, error bar = SEM, **p < 0.01, ***p < 0.001).

**Figure 5.**
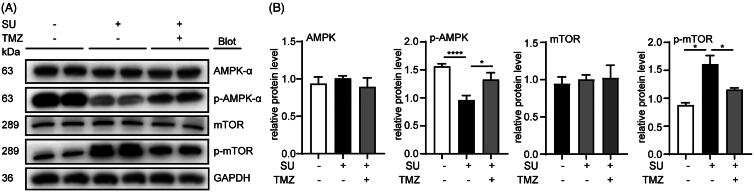
SU-induced AMPK/mTOR activation inhibition in H9c2 cardiomyocytes is reversed by TMZ. (A, B) Western blots of AMPK/mTOR pathway in vehicle-, SU- and SU-TMZ-treated H9c2 cardiomyocytes and statistical analysis (*n* = 5, 6, 4, 4, respectively). (The Student’s two-tailed *t*-test was used, error bar = SEM, *p < 0.05, ****p < 0.0001).

Having identified the AMPK/mTOR pathway as one major pathway involved in SU-induced autophagic inhibition, we next examined whether TMZ regulated SU-induced autophagy via the same pathway. Using western blot, we examined AMPK, p-AMPK, mTOR and p-mTOR levels in both SU- and SU-TMZ-treated mouse cardiac homogenates and H9c2 cells. We found that although TMZ did not affect total protein levels of AMPK and mTOR, it reversed SU-induced AMPK inactivation and mTOR activation in both cardiac homogenates (Figure 4(A,B)) and H9c2 cells (Figure 5(A,B)). These results show that TMZ alleviates SU-induced autophagy via the AMPK/mTOR pathway both *in vivo* and *in vitro*.

## Discussion

Potential cardiotoxicity of anticancer chemotherapies continue to hinder their clinical application and impair life quality of cancer fighters and cancer survivors. Developing prevention and treatment strategies for chemotherapy-induced cardiotoxicity is urgent to improve outlook and overall prognosis and survival of cancer patients. Sunitinib is a tyrosine kinase inhibitor with a small incidence of undesired cardiovascular events, including hypertension and LVEF decline. TMZ has been reported to be cardioprotective in a range of cardiovascular conditions, but its effects in SU-induced cardiotoxicity remain unknown. In the present study, we show that TMZ is able to reverse SU-induced hypertension and LVD, with the involvement of AMPK/mTOR/autophagy pathway in cardiomyocytes. These results provide an avenue for developing molecular and cellular treatment strategies for SU-induced cardiotoxicity.

Cardiovascular adverse effects of SU commonly include hypertension, LVEF declines, and congestive heart failure (Chu et al. 2007). Initially designed as a selective tyrosine kinase inhibitor, SU primarily inhibits vascular endothelial cell growth factor receptors (VEGFRs) 1-3, platelet-derived growth factor receptors (PDGRF) α and β, FMS-like tyrosine kinase-3 (Flt-3), the stem cell factor receptor c-kit, colony-stimulating factor 1 receptor (CSF1R), and the ret oncogene product, RET (Chu et al. 2007), as molecular targets. However, recent kinome and transcriptome profiling revealed that cytotoxic effects of SU are not limited to inhibition of these targets, but are instead far more broad-targeted (Stuhlmiller et al. 2017). Cardiomyocytes constitute the cellular component of the myocardium and are highly energy-consumptive and sensitive to SU cytotoxicities. Recent studies revealed the mechanisms of cardiomyocyte toxicities of SU to include mitochondrial dysfunction (Varga et al. 2015), hypertrophy influenced by mitogen-activated protein kinases pathways and aryl hydrocarbon receptor signaling pathway (Maayah et al. 2014; Korashy et al. 2015), autophagy (Zhao et al. 2010), and AMP-activated protein kinase inhibition (Kerkela et al. 2009). Cardioprotective strategies should, therefore, be designed to counteract these mechanisms of cardiotoxicity.

TMZ is a drug developed to treat angina pectoris. The primary molecular inhibition target of TMZ is HADHA, through which TMZ switches myocardial energy metabolism from β-oxidation of fatty acids to the faster and more efficient glucose utilization, supplying the heart with a more proficient energy turnover, making the heart more durable under conditions of energy shortage such as ischemia, diabetic cardiomyopathy or drug toxicity (Chrusciel et al. 2014; Lopatin et al. 2016). It has been previously reported that TMZ regulates autophagy, and this regulation could either promote or inhibit, depending on the context. TMZ enhanced autophagy steatotic liver graft preservation (Zaouali et al. 2013), skeletal muscle myotubes under stress-induced atrophy (Ferraro et al. 2013) and diabetic cardiomyopathy (Zhang et al. 2016), and inhibited autophagy in myocardial ischemia/reperfusion injury (Wu et al. 2018). Because autophagy has been identified as a key mechanism of SU cardiotoxicity, it could be speculated that TMZ may be able to regulate SU cardiotoxicity via autophagy-associated mechanisms. Our results present for the first time that TMZ possesses potent cardioprotective effects against SU cardiotoxicity via the upregulation of AMPK/mTOR/autophagy machineries. Although this finding does not exclude that other existing pathways may also influence alleviating effects of TMZ in SU cardiotoxicity, it provides foundation for further development of treatments against SU-induced cardiotoxicity.

Apart from autophagic machinery, we also eliminated the possibility that SU-induced cardiomyocyte death was mediated by apoptosis. We found using TUNEL assay that SU increased rate of TUNEL-positive cells in mouse hearts, but in later *in vitro* western blot analysis, we found no alterations of apoptotic markers. TUNEL assay is a common detection method for apoptosis, which detects DNA strand breaks as a marker for apoptosis. However, the DNA strand break is not specifically a product of apoptosis (Gál et al. 2000). It is not to be excluded that our results of TUNEL-positivity may be elicited by mechanisms other than apoptosis which ended up in cell death.

Although TMZ may be cardioprotective against SU cardiotoxicity, one main problem remains undefined by our study: whether protective effects of TMZ compromise anticancer efficacy of SU. To date, there are few studies reporting the effects of TMZ in cancer (Molinari et al. 2017; Halama et al. 2018). To validate TMZ as a safe drug for SU-receiving patients, further investigations are required to exclude the possibility that TMZ may undermine SU anticancer effects.

Conclusively, the present study shows that SU induces hypertension and left ventricular dysfunction in mice and cell viability loss in H9c2 cardiomyocytes via inhibition of AMPK/mTOR/autophagy pathway; trimetazidine is able to reverse sunitinib-induced cardiotoxicity in mice as well as viability loss in H9c2 cardiomyocytes via involvement of AMPK/mTOR/autophagy pathway activation.

## Supplementary Material

supplementary_figure_caption.docx

supplementary_figure_1_-_apoptotic_markers.tif
