# Supplementary material for: Trimetazidine ameliorates sunitinib-induced cardiotoxicity in mice via the AMPK/mTOR/autophagy pathway
Source: Pharm Biol. 2019 Sep 23;57(1):625–31. doi: 10.1080/13880209.2019.1657905 (PMC6764339; doi:10.1080/13880209.2019.1657905)
Supplement: supplementary_figure_caption.docx [file IPHB_A_1657905_SM7345.docx]

**Supplementary figure caption**

Figure S1 SU does not activate apoptotic markers in H9C2 cardiomyocytes. A.B. Western blots of apoptotic markers in vehicle- and SU-treated H9C2 cardiomyocytes and statistical analysis (n = 4). (The Student’s two-tailed *t*-test was used, error bar = SEM)
