# Supplementary figures and images for: Trimetazidine ameliorates sunitinib-induced cardiotoxicity in mice via the AMPK/mTOR/autophagy pathway
Source: Pharm Biol. 2019 Sep 23;57(1):625–31. doi: 10.1080/13880209.2019.1657905 (PMC6764339; doi:10.1080/13880209.2019.1657905)

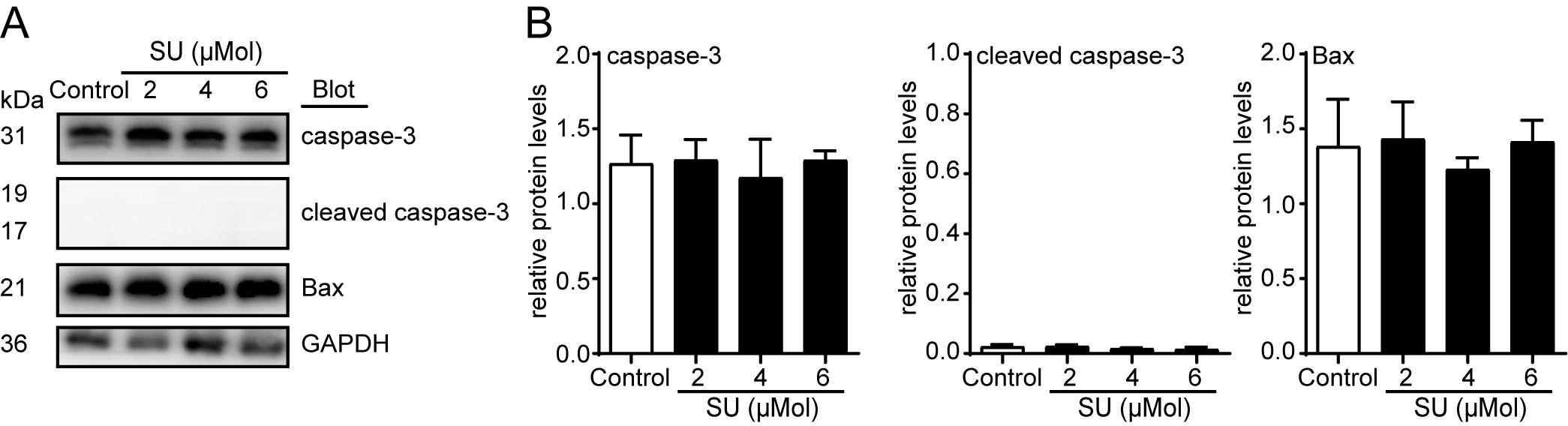

Supplement: supplementary_figure_1_-_apoptotic_markers.tif [file IPHB_A_1657905_SM7344.tif]
